# Valvular Lesions of the Heart, Commonly Known as Cardiac Murmurs*Read before Atlanta Society of Medicine, February 16, 1899.

**Published:** 1899-03

**Authors:** J. L. Campbell

**Affiliations:** Atlanta, Ga.


### VALVULAR LESIONS OF THE HEART, COMMONLY KNOWN AS CARDIAC MURMURS.*

By J. L. CAMPBELL, M.D.,

Atlanta, Ga.

"Cardiac murmurs are adventitious sounds heard in connection with the heart in addition to, or in place of, those sounds that exist in health." (*Page.*)

"In relation to their seat of generation they are designated as mitral, aortic, pulmonary, and tricuspid. According to the period of the heart's cycle at which they occur, they are divided into

*Read before Atlanta Society of Medicine, February 16, 1899.

systolic, those occurring during the systole; diastolic, those occurring in diastole; presystolic and diastolic, those occurring just before systole and diastole respectively." (*Gould.*)

Cardio-respiratory murmurs are caused by the impact of the heart against the lungs.

*Etiology* depends on the location and character. In the vast majority of cases it is rheumatic endocarditis. The disease may come on slowly, or may be due to repeated acute attacks. The aortic orifice may be affected from sclerosis of the valves, due to hard physical labor or interstitial nephritis. The lesions may result from endocarditis following any of the acute febrile diseases, and after tonsillitis. In a number of the cases I have seen, the only history traceable was repeated sore throats which may have been syphilitic in character. Gonorrhea and pyemia also play important parts. It may come on independently of all preexisting diseases. Endocarditis of the adult is always on the left side, while in the fetus it is on the right.

We may have insufficiency of the valves at the mitral or tricuspid orifices, due to dilatation of the ventricles, and known as relative insufficiency.

Bright's disease, lead poisoning, syphilis, and chronic alcoholism are all important factors in aortic lesions.

According to Page we have the lesions occurring as follows: (1) Most frequently mitral regurgitation, (2) aortic obstruction, (3) aortic regurgitation, (4) mitral obstruction, (5) tricuspid regurgitation—relative, (6) pulmonary obstruction—most frequent inflammatory condition of the heart, (7) pulmonary regurgitation—very rare, and (8) tricuspid obstruction—hardly known.

These lesions are often combined. It is hardly necessary to tabulate the manner in which these occur, as we may have almost any two or more combined. I have seen aortic obstruction and regurgitation and mitral regurgitation combined most frequently.

*Morbid Anatomy.*—In the mitral valve there may be only a slight thickening of the edges, or there may be calcareous deposits of only limited areas to almost complete destruction of one segment, or thickening to such an extent as to make the valve almost immovable. There may be vegetations and ulcerations, as are frequently found in gonorrheal endocarditis and others of infectious nature.

*Mitral Obstruction.*—The auricular-ventricular opening may be only slightly narrowed, as by adhesions of the edges of the valves; or it may be narrowed to such an extent that only a small probe can be introduced.

*Tricuspid Valves.*—There are sometimes vegetations on the tricuspid valves, as we have seen in one case where the patient died suddenly, probably, due to the dislodgment of one of these forming an embolus in one of the pulmonary arteries.

In aortic lesions, we have seen great variations extending all the way from slight thickening around the corpus arantii to thickening of the valves, to such an extent that they were almost immovable. There is generally a calcareous deposit on the inner side of the aorta, extending for an inch or two above the valves. We saw in one case a perforation of one segment of the semi-luna valve, and in another case the posterior segment of the valve was so short that it was with difficulty we could get the handle of the knife between it and the wall of the artery.

In the aortic obstructive cases that we have seen, the narrowing was largely due to the atheromatous condition of the valves not allowing them to fit snugly against the side of the vessel during the ventricular systole.

Cardiac hypertrophy takes place in mitral obstruction in the following manner: First, of the left auricle, due to overwork in forcing the blood through the narrow orifice; next, in the right ventricle, to overcome the damming back of the blood on the pulmonary circulation.

In mitral regurgitation, the enlargement occurs to a limited extent first in the left auricle, because it has to handle part of the blood twice, and also in the left ventricle for the same reason, plus the effort to send the normal amount of blood through the aortic opening. Finally, we have hypertrophy of the right ventricle, due to the passive congestion in the lungs and the effort of that side to overcome it.

In aortic obstruction, or regurgitation, we have the greatest amount of cardiac hypertrophy, because here we have a part of the heart handling the blood, that is capable of great increase in size, viz.: the left ventricle. The increase in size is about equal in either of these conditions; as in the first, the left ventricle has to overcome an obstruction and force the blood through a narrow orifice. In the regurgitation it has an increased quantity to force through the normal orifice. The hypertrophy is most marked where these lesions occur together, and goes to such an extent that the apex beat is displaced from one and a half, two or three inches to the left and downward. In all these lesions the murmur does not indicate the amount of involvement, as in some of the loudest murmurs I ever heard we found only a small calcareous deposit on the valves and a slight thickening of the aorta.

*Other Organs.*—The lungs are the first organs involved outside the heart, being the nearest and most concerned in the circulation. As soon as dilatation takes the place of hypertrophy the blood is dammed back on the lungs, the air vesicles are encroached upon by the distended capillaries, and the exudation of serum in them causes less breathing space, and consequently diminished aeration of the blood. In short, the lungs are in a state of marked passive congestion, and in a patient dead from this disease, the bloody serum flows quite freely from the cut surfaces.

The liver suffers next, and as dilatation takes place, we frequently have marked enlargement of this organ, extending, as I have seen in one or two cases, even down into the pelvis. This enlargement is due to passive congestion. If compensation is again established, we have a formation of new connective tissue and a contraction of the organ. This is occurring in a case I now have under observation, and he has ascites to a marked extent. The spleen is also enlarged, and the mucous membrane of the whole intestinal canal is passively congested.

The kidneys suffer with the other organs, and there is a cloudy swelling or sometimes interstitial nephritis. There may or may not be albumen in the urine. In repeated examinations I have failed to find it, even when there is general anasarca, and only a small amount of the urine voided. Osler says that albumen is one of the common symptoms of loss of compensation, and it may be present, but we have failed to find it in a number of cases.

*Symptoms.*—Aortic regurgitation and stenosis present the fewest symptoms, and Osler says the condition may be discovered accidentally in persons who have not presented any feature of cardiac disease.

Lesions of the mitral valve give us the longest train of symptoms. When compensation begins to give way, the first thing we are consulted for is shortness of breath. This is due to the passive congestion of the lungs. There may be at the same time, or shortly after, a swelling of the feet, gradually extending up the leg as the disease progresses.

There is nearly always a cough, accompanied at first by white frothy expectoration, becoming dark and sometimes streaked with blood as the end approaches.

The patient is unable to lie flat down in bed, using frequently three or four pillows, and sometimes can only rest sitting in a chair or in a kneeling position. The appetite is usually poor. The stomach presents more or less catarrhal symptoms, sometimes those of dyspepsia. The patient may come for that condition, complaining of indigestion. The bowels may be loose, or more frequently constipated. Patients complain of palpitation and pain in the region of the heart, but these are rare except in aortic lesions with marked artero-sclerosis. There is frequent pain in the epigastric region, especially when there is enlargement of the liver.

The pulse is usually regular and little can be gained from it, except in advanced cases. In aortic regurgitation, we have the Corigan, or water-hammer pulse, which is typical of the lesion, also the blush in the finger-nails may be present. When compensation is complete, none, or few of these symptoms are present, only on extreme exertion, and they disappear to some extent when it is reestablished.

*Physical Signs—Inspection.*—The apex of the heart will be seen, if seen at all, displaced downward and outward, in mitral regurgitation and aortic murmurs. There is no displacement in mitral obstruction. The displacement is due to hypertrophy of the left ventricle.

In extreme cases, the face will be drawn and the muscles of the neck contracted. The superficial veins will be distended, due to passive congestion. When there is dilatation of the right ventricle, epigastric pulsation will be seen.

It is said that in mitral obstruction, pulsation is seen about the third interspace to the left of the sternum, due to hypertrophy of the left auricle.

*Palpation.*—It may not be possible to see the apex beat. Then, in the cases we have mentioned, it can be felt to the left and downward. In mitral obstruction, there is a distinct thrill felt at the apex. Thrills are also present in aortic obstruction, and felt at the second interspace to the right of the sternum. The heart's impulse against the chest wall is forcible while hypertrophy lasts, but diffuse and weak during dilation.

*Percussion.*—Percussion shows increase in the area of cardiac dullness more transversely than vertically and must not be mistaken for pericardial effusions.

*Auscultation.*—Murmurs at the mitral valve are heard at the apex, tricuspid, at the ensiform cartilage, aortic, at the second interspace to the right of the sternum, pulmonary, at the second interspace to the left of the sternum.

A murmur heard at the apex, systolic in time and blowing in quality, and conducted to the axilla, and heard posteriorly at the lower angle of the scapula, is a mitral regurgitant. One heard at the ensiform cartilage is connected with the tricuspid valve. Those heard at the second interspace to the right of the sternum are aortic, and, if systolic in time, accompanied by an intense thrill and conducted into the vessels of the neck, is aortic obstruction. One heard at the same point, but blowing in character and diastolic in time, conducted both up and down the sternum, is an aortic regurgitant.

Pulmonary murmurs are rare, therefore not necessary to be discussed.

Double aortic murmurs are very common and are both diastolic and systolic in time, and are steam-tug in character. Murmurs may be so loud that they can be heard at any point on the chest, but usually the maximum point of intensity is at the parts above named.

*Treatment.*—If we can so combine our remedies that we can get only a moderate amount of arterial tension, increase the elimination of the urine, and the action of the bowels, and at the same time stimulate the contractions of the ventricles, we have accomplished our purpose and have placed our patient under the most favorable conditions possible.

Rest is one of the best methods of giving our patient the greatest amount of comfort and reestablishing compensation.

